# Inverse Finite Element Approach to Identify the Post-Necking Hardening Behavior of Polyamide 12 under Uniaxial Tension

**DOI:** 10.3390/polym14173476

**Published:** 2022-08-25

**Authors:** Cornelia Amstutz, Bernhard Weisse, Andreas Haeberlin, Jürgen Burger, Adrian Zurbuchen

**Affiliations:** 1 School of Biomedical and Precision Engineering, University of Bern, 3008 Bern, Switzerland; 2 EMPA, Swiss Federal Laboratories for Material Science and Technology, Mechanical Systems Engineering, 8600 Duebendorf, Switzerland; 3 Department of Cardiology, Inselspital, Bern University Hospital, University of Bern, 3010 Bern, Switzerland

**Keywords:** PA12, mechanical properties, inverse identification, post-necking, modeling, plasticity

## Abstract

Finite-element (FE) simulations that go beyond the linear elastic limit of materials can aid the development of polymeric products such as stretch blow molded angioplasty balloons. The FE model requires the input of an appropriate elastoplastic material model. Up to the onset of necking, the identification of the hardening curve is well established. Subsequently, additional information such as the cross-section and the triaxial stress state inside the specimen is required. The present study aims to inversely identify the post-necking hardening behavior of the semi-crystalline polymer polyamide 12 (PA12) at different temperatures. Our approach uses structural FE simulations of a dog-bone tensile specimen in LS-DYNA with mesh sizes of 1 mm and 2 mm, respectively. The FE simulations are coupled with an optimization routine defined in LS-OPT to identify material properties matching the experimental behavior. A Von Mises yield criterion coupled with a user-defined hardening curve (HC) were considered. Up to the beginning of necking, the Hockett–Sherby hardening law achieved the best fit to the experimental HC. To fit the entire HC until fracture, an extension of the Hockett–Sherby law with power-law functions achieved an excellent fit. Comparing the simulation and the experiment, the following coefficient of determination $R^{2}$ could be achieved: Group I: $R^{2}$ > 0.9743; Group II: $R^{2}$ > 0.9653; Group III: $R^{2}$ > 0.9927. Using an inverse approach, we were able to determine the deformation behavior of PA12 under uniaxial tension for different temperatures and mathematically describe the HC.

## 1. Introduction

Polymeric materials have become an integral part of many industrial fields and must fulfill structural requirements. To avoid expensive trials in the design process phase of polymeric products, structural simulations based on the finite-element (FE) method are used. FE simulation tools require the input of appropriate mechanical material properties. The linear elastic material model requires the input of the Young’s modulus as well as the Poisson’s ratio. Both values are available for a broad range of materials. Due to the lack of further data, many numerical simulations do not include plasticity and are limited to linear elasticity. However, for example, the injection stretch blow molding of percutaneous transluminal coronary angioplasty (PTCA) balloons requires the consideration of plasticity as the stretching load during the manufacturing process goes over the elastic limit of the material [1]. The plastic theory requires additional information, namely, a yield function, a flow-rule, and the definition of a hardening curve. The extraction of the Young’s modulus, the Poisson’s ratio, and the hardening curve up to the onset of necking is well established. However, most of the available models have been developed for metals, which mostly fracture after necking. Polymers, on the other hand, often show a neck propagation followed by a strain hardening before fracture [2].

Post-necking or large strain information can either be obtained from extracting additional information from the uniaxial tensile tests or from different experimental tests such as hydraulic bulge tests or cylinder or stacked compression [3,4,5]. Additional information can be obtained from full-field strain measurements with digital image correlation (DIC) [6] or from the measurement of the cross-section with video or clip-on extensometers during the experiment. However, full-field DIC is often not available, and the measurement of the cross-section requires the knowledge of the exact location where the neck forms. This is, depending on the specimen geometry, not known prior to testing. Furthermore, neither of these tests are able to account for the triaxial stress state inside the neck [7,8], and therefore subsequent corrections are necessary. 

In the past, large strain material behaviors have been studied extensively for semi-crystalline polymers such as high-density polyethylene (HDPE), polyvinyl chloride (PVC), and polycarbonate (PC) [8,9,10,11,12]. Most of those studies are based on the measurement of the cross-sectional change during necking. Arriaga et al. [13] proposed a method that does not rely on measurements of the cross-section but uses some general mathematical conversion methods on the hardening curve obtained from simple uniaxial tensile tests. 

Another possibility to obtain large strain material properties is the inverse identification FE approach. This approach uses FE simulations coupled with an optimization routine to identify the material properties that represent best the experimentally determined mechanical behavior of a specimen. Shin et al. [14] used an inverse finite-element (FE) approach to fit the hardening law to the experimental data of fiber-reinforced polymers without necking. Zhang et al. [15] proposed an inverse approach synchronized with a DIC setup to identify the post-necking behavior of metallic sheets, without neck propagation. To the authors’ best knowledge, no method is publicly available to obtain the hardening curve for polymers with neck propagation and strain hardening based on experimental force–displacement curves without manual trial and error methods. Furthermore, the inverse identification approach on the post-necking properties of semi-crystalline polymers has to our knowledge not been studied yet.

The semi-crystalline polymer Polyamide 12 (PA12) has good chemical and abrasion resistance as well as a low coefficient of friction. In addition, its high strength combined with high compliance [16] makes it a widely used polymer in many industrial sectors such as automotive, electronics, packaging, and medical devices. The most common processing techniques to manufacture PA12 are injection molding, pipe extrusion, film extrusion, and blow-molding. PA12 was selected for this work due to its frequent use in the manufacturing of PTCA balloons [17] and PTCA catheter shafts.

The present work inversely identifies the hardening curves post-necking for the semi-crystalline polymer PA12 in a temperature range from 23 °C to 100 °C. The used experimental data are based on uniaxial tensile tests on dog bone specimens published by Amstutz et al. [18].

## 2. Materials and Methods

In [18], uniaxial tensile tests were performed on ambient (stored at 23 °C and 50% r. H.) and conditioned (stored in water at 23 °C for 40 days) injection molded Grilamid® L25 PA12 dog bone specimens (ISO 527-2, Type 1A, EMS-CHEMIE AG, Domat/Ems, Switzerland). The specimens had a cross-section of 10 × 4 mm^2^ and a total length of 170 mm and were subjected to a tensile load with a constant cross-head velocity of 50 mm/min until fracture. The tests were performed at 23 °C, 37 °C, 50 °C, 80 °C, and 100 °C. This temperature range was adopted for the present work since it covers the temperatures used during the manufacturing steps of PTCA balloons [1] and shafts. 

The force–displacement curves, the identified Young’s modulus (E) and the Poisson’s ratio (ν) were used from [18] for the present work. 

The behavior of the force–displacement curves were divided in three groups:

- Necking followed by stable neck propagation until fracture (23 °C ambient, conditioned) (Group I);
- Necking followed by strain hardening (37 °C ambient and conditioned, 50 °C ambient) (Group II);
- Without necking (50 °C conditioned, 80 °C ambient and conditioned, 100 °C) (Group III).

### 2.1. Stress and Strain Conversion

**Figure 1:**
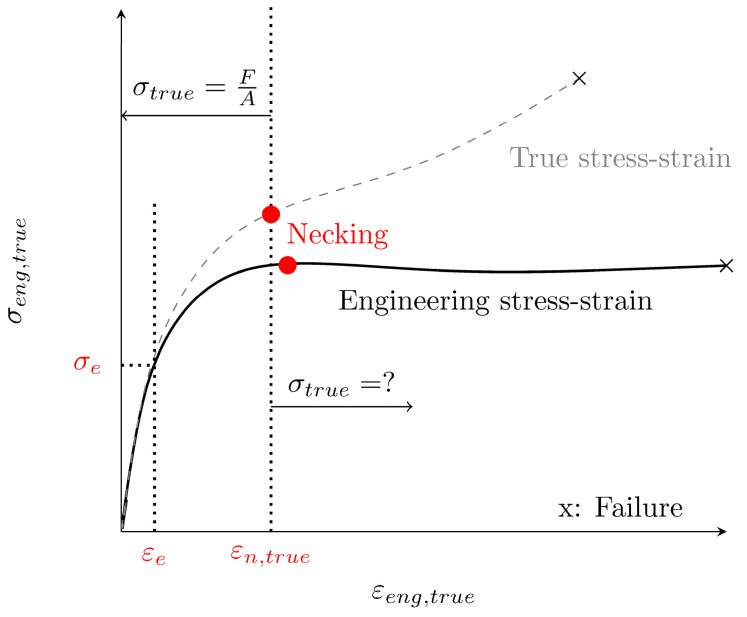
Exemplary engineering stress–strain and true stress–strain curve of a semi-crystalline thermoplastic material. The point of necking is defined as the highest engineering stress followed by a stress decline. $\varepsilon_{e}$ marks the end of the elasticity and the true strain $\varepsilon_{n,true}$ the beginning of the necking.

The engineering stress–strain curve (cf. Figure 1) depends on the applied force F and the initial cross section $A_{0}$. Therefore, the engineering stress $\sigma_{eng}$ starts to decrease after the sample starts to neck locally. Nevertheless, the true stress inside the specimen depends on the actual cross-section A and increases after necking since the cross-section locally reduces. Based on the assumptions that (a) the stress distribution along the gauge section of the specimen is uniaxial and (b) uniform, and that (c) the material behaves in an incompressible manner, $\sigma_{true}$ and $\varepsilon_{true}$ can be approximated by Equations (1) and (2), respectively.

(1) $$\sigma_{true}=\frac{F}{A}=\sigma_{eng}\cdot \left(1+\varepsilon_{eng}\right)\;\left[\mathrm{MPa}\right]$$

(2) $$\varepsilon_{true}=\int_{L_{0}}^{L}\frac{∆L}{L}=\ln\left(1+\varepsilon_{eng}\right)\;\left[-\right]$$

While this approximation of $\sigma_{true}$ and $\varepsilon_{true}$ is valid until the specimen starts to neck locally, the assumptions (a, b) are not fulfilled anymore after necking. As described in [3,4], the stress distribution changes to a triaxial stress state in the region of the neck, and therefore the stress is neither uniaxial nor uniform anymore. To identify the mechanical properties after necking, an inverse identification approach was used and is explained in Section 2.2.2.

After reaching the elastic limit $\sigma_{e}\;\left(\varepsilon_{e}\right)$ (cf. Figure 1), plastic effects must be considered. The effective plastic strain $\varepsilon_{pl}$ can be obtained from the true stress–strain using Equation (3).

(3) $$\varepsilon_{pl}=\varepsilon_{true}-\frac{\sigma_{true}}{E}\;\left[-\right].$$

The hardening curve (HC) is defined by $\varepsilon_{pl}$ and $\sigma_{true}$ and goes from $\sigma_{e}$ until failure. Therefore, $\sigma_{e}$ is the first true stress value of the HC, where $\varepsilon_{pl}=0$.

To obtain a temperature-dependent Young’s modulus E and proportionality limit $\sigma_{e}$, the model of Mahieux et al. (cf. Equation (4)) [19,20] was used. *P* represents either E or $\sigma_{e}$.

(4) $$P\left(T\right)=\left(P_{g}-P_{r}\right)*e^{-{\left(\frac{T}{T_{g}}\right)}^{m}}+P_{r}*e^{-{\left(\frac{T}{T_{d}}\right)}^{n}}\;\left[\mathrm{MPa}\right].$$

As a measure of how well the model fits the experimental data, the coefficient of determination $R^{2}$ was evaluated and rated as:

$R^{2}$ = 0: no fit
$R^{2}$ = 1: perfect fit
0 < $R^{2}$ < 1: partial correct fit

### 2.2. Elastoplastic Constitutive Model and Inverse Identification Approach

The linear part of the stress–strain curve is described by Hook’s Law (cf. Equation (5)).

(5) $$\sigma =E\cdot \varepsilon_{el}\;\left[\mathrm{MPa}\right].$$

To introduce plasticity to the material model, a yield criterion is required. In the present work, an isotropic elastoplastic model was considered using the Von Mises yield criterion *Y* (cf. Equation (6)), where $J_{2}$ is the second invariant of the deviatoric stress tensor, and $\sigma_{h}$ is the yield strength defined by the isotropic hardening model. This criterion states that a material starts yielding once the Von Mises stress $\sigma_{VM}$ in the material exceeds $\sigma_{h}$.

(6) $$Y\left(J_{2}\right)=\underset{\sigma_{VM}}{\underbrace{\sqrt{3\cdot J_{2}}}}-\sigma_{h}\left(\varepsilon_{pl}\right)\;\left[\mathrm{MPa}\right].$$

#### 2.2.1. Hardening Model until Necking

Until the onset of necking, the HC is known and can be extracted directly from the experiment using Equations (1)–(3). The following power-law (cf. Equations (7)–(9)) [21,22,23] and exponential (cf. Equations (10) and (11)) [24,25] models are used to describe the isotropic hardening $\sigma_{h}$ prior to necking.

(7) $$\sigma_{Ludwik}=\sigma_{e}+k\cdot \varepsilon_{pl}^{n}\;\left[\mathrm{MPa}\right],$$

(8) $$\sigma_{Swift}=\sigma_{e}\cdot {\left(1+\frac{\varepsilon_{pl}}{\varepsilon_{0}}\right)}^{n}\;\left[\mathrm{MPa}\right],\;$$

(9) $$\sigma_{Ghosh}=\sigma_{e}+k\cdot {\left(\varepsilon_{0}+\varepsilon_{pl}\right)}^{n}\;\left[\mathrm{MPa}\right],$$

(10) $$\sigma_{Voce}=\sigma_{e}+\sigma_{\infty }\cdot \left(1-e^{-k\cdot \varepsilon_{pl}}\right)\;\left[\mathrm{MPa}\right],$$

(11) $$\sigma_{Hockett-Sherby}=\sigma_{e}+\left(\sigma_{\infty }-\sigma_{e}\right)\left(1-e^{-k\cdot \varepsilon_{pl}^{n}}\right)\left[\mathrm{MPa}\right].$$

The specific material parameters $\sigma_{e}$, k, n, $\varepsilon_{0}$, and $\sigma_{\infty }$ are derived from a least squares curve fitted to the experimental HC. The quality of the fit is assessed by $R^{2}$. 

#### 2.2.2. Hardening Model Post Necking

As mentioned above, after the onset of necking, the HC can no longer be extracted from the experimental data. Therefore, the approach from Section 2.2.1 is not valid anymore, and the HC is unknown. After the specimen starts to neck more information, the actual cross-section of the specimen is needed. As Kwon et al. [8] showed, further conversion of the data is required, since the stress is no longer uniaxial and uniform but triaxial and localized in the neck. This triaxial stress state influences the Von Mises stress, since it is no longer solely based on the stress in the tensile direction. 

**Figure 2:**
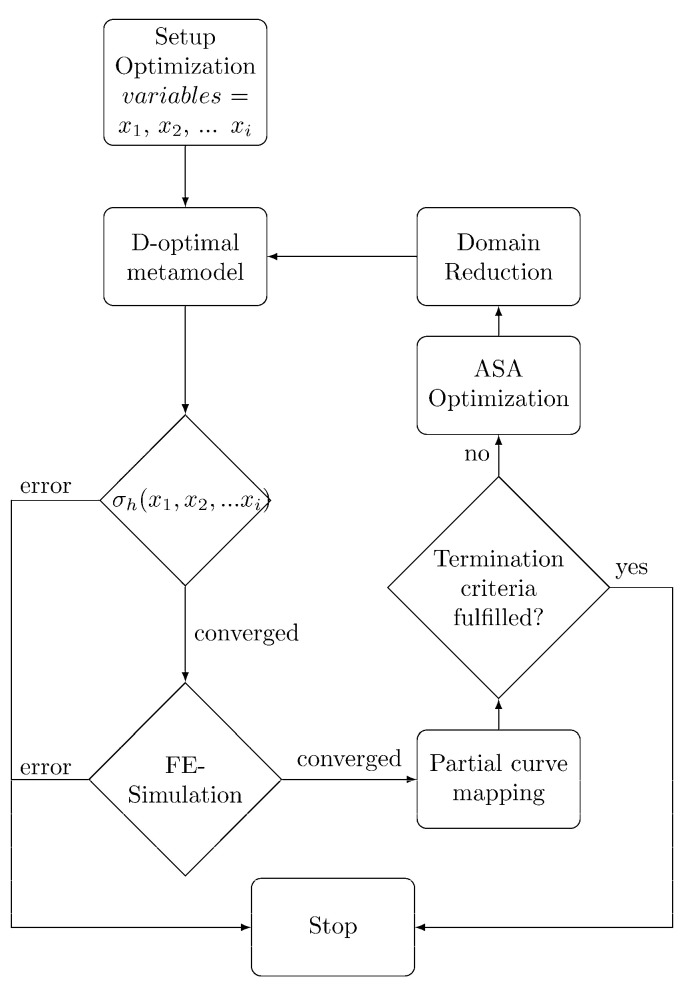
Optimization routine in LS-OPT 6.0.0 using an FE simulation in LS-DYNA of the dog bone tensile specimen and a user-defined HC $\sigma_{h}$ to minimize the difference between the simulated and the experimental force–displacement curve.

Since the HC was unknown, an inverse identification approach was used. This approach used the optimization tool LS-OPT 6.0.0 (Livermore Software Technology Corporation, Livermore, CA, USA) to optimize the HC used in an FE simulation to match the experimental force–displacement curve. The optimization routine is shown in Figure 2.

In the first step, the fitting parameters ($x_{1},x_{2},\ldots x_{i}$) are defined as variables. 

Based on those variables, a user-defined HC $\sigma_{h}$ is calculated using Python 3.8 with either polynomial (cf. Equation (12)) or exponential (cf. Equation (13)) functions.

(12) $$\sigma_{h}\left(x_{1},\;x_{2},\;x_{3}\right)=x_{1}\cdot {\left(\varepsilon -\varepsilon_{i}\right)}^{3}+x_{2}\cdot {\left(\varepsilon -\varepsilon_{i}\right)}^{2}+x_{3}\cdot \left(\varepsilon -\varepsilon_{i}\right),$$

(13) $$\sigma_{h}\left(x_{1}\right)=\sigma_{i}\cdot e^{x_{1}\cdot {\left(\varepsilon -\varepsilon_{i}\right)}^{x_{2}}}.\;$$

Because of the tendency to oscillate (Runge’s phenomenon), higher order polynomials were not considered. For the fitting parameters, ranges of 0 to 500 and 0 to 10 were used for Equations (12) and (13), respectively. In these ranges, the experimental design space was defined by a linear D-optimal metamodel [26] with 16 points. Due to the complexity of the HC, it was not possible to fit the whole HC with only one polynomial or exponential function. To avoid instability of the FE simulations in the large strain region due to a bad fit, the HC was divided into various strain regions, which were then fitted successively. Therefore, $\varepsilon_{i}$ and $\sigma_{i}$ defined the last point of the already-fitted region.

The HC $\sigma_{h}$ was used in the piecewise linear plasticity material model (MAT024) of a FE simulation of the dog bone specimen using the implicit solver of LS-DYNA (Livermore Software Technology Corporation, Livermore, CA, USA) smp dR11.1. 

The calculated force–displacement curve was then compared to the experimental curve by curve mapping. An Adaptive Simulated Annealing (ASA) optimization algorithm was used to minimize the difference between the curves [27,28]. Based on the result of the optimization, the parameter range was reduced, and a new design space was defined. A maximum of 20 optimization iterations was performed. 

**Figure 3:**
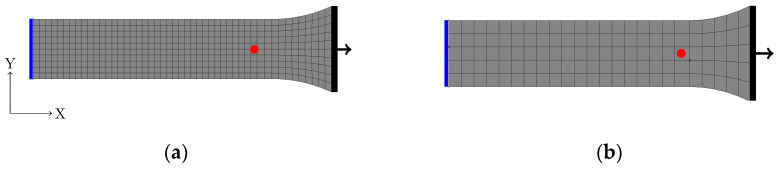
A 3D FE model of the dog bone specimen (ISO 527-2, Type 1A with a total length of 170 mm and a cross section of 10 × 4 mm^2^) using symmetry in the x-direction. The left-hand side of the specimen (blue) is fixed, and the right-hand side (black) is subjected to a displacement with kinematic coupling functionality. The red point, which is 35 mm from the symmetry plane, indicates the point where local displacement is measured. (**a**) MS = 1 mm (1960 elements); (**b**) MS = 2 mm (250 elements).

**Figure A1:**
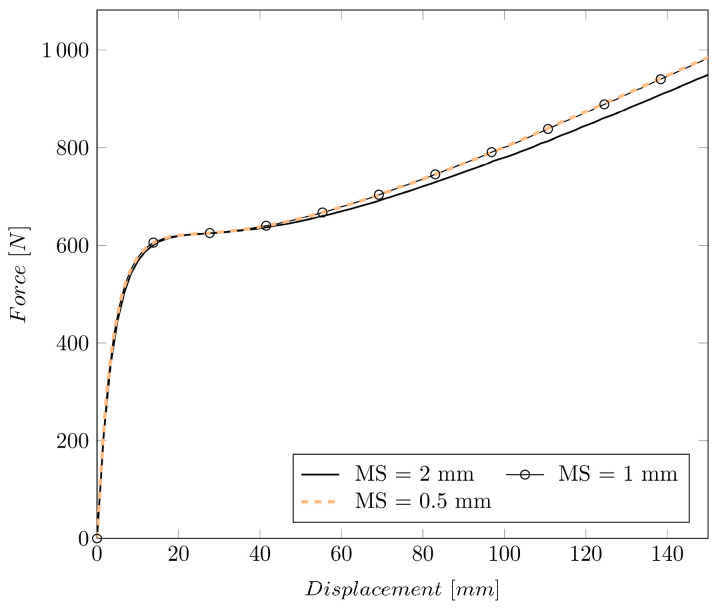
Comparison of the force–displacement curves of uniaxial tensile tests at 100 °C for a mesh size of 0.5 mm, 1 mm, and 2 mm.

According to [26], after necking, the obtained HC depends on the mesh size (MS) [29]. Therefore, two 3-dimensional (3D) FE models with different mesh sizes (1 mm and 2 mm) were generated (cf. Figure 3). To ensure that a mesh size of 1 mm could converge, one additional simulation was performed with a mesh size of 0.5 mm. The results are shown in Appendix A Figure A1. Both mesh sizes (0.5 mm and 1 mm) showed the same result. 

To reduce the computational effort, the symmetry of the specimen in the x-direction was used. Further symmetries were not considered to avoid influences on the neck shape and formation, respectively. On the symmetry plane of the model (blue line), the x-direction as well as the rotations around x, y, and z-axes were fixed. A displacement in the x-direction was applied on the right side (black line) using the kinematic coupling functionality (with a reference node). In this model, the reaction force F was evaluated at the nodes on the symmetry plane, and the displacement l was measured at the red point in the same place as in the experiment (cf. Figure 3). 

Based on the identified hardening curves, a single mathematical expression was derived to express the entire HC.

## 3. Results

### 3.1. Linear Elastic Values 

An overview of the linear elastic values can be found in Table 1. 

The resulting variables of the temperature-dependent linear elastic values fitted to Equation (4) are shown in Table 2. 

**Figure 4:**
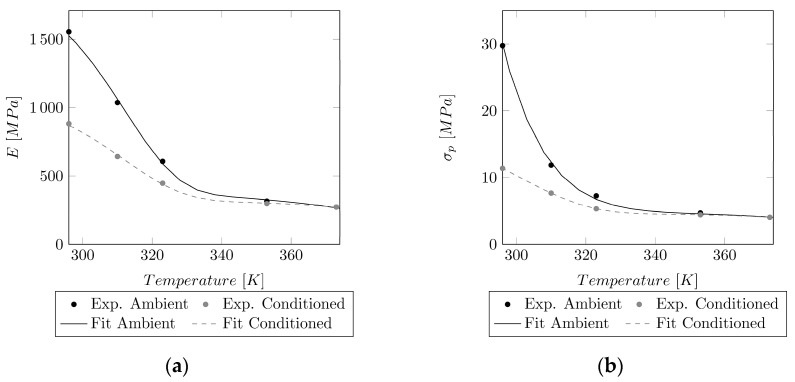
Comparison of the model to the ambient and conditioned experimental linear elastic values in a temperature range of 23–100 °C (296–373 K). (**a**) Temperature-dependent model of Mahieux et al. and experimental values of the experimental Young’s modulus E. (**b**) Temperature-dependent model of Mahieux et al. and values of the experimental proportionality limit $\sigma_{p}$.

The results of the fit are depicted in Figure 4. 

### 3.2. Curve Fitting until Necking

The HC for Group I and II until necking was extracted using Equations (1)–(3). Overall, the best fit for the HC until necking was achieved with the Hockett–Sherby model (cf. Table 3). Only the variables of the best fit are shown. The results of the fit to Ludwik, Swift, Ghosh, and Voce can be found in the Appendix B Table A1. Since this material model is only applicable until necking starts, for each model the maximum valid Von Mises Stress $\sigma_{VM}$ is given in Table 3. 

As mentioned before, Group III showed no necking. Therefore, the entire HC could be extracted from the experimental data. However, the behavior of these curves was too complex to be described with the available laws (Equations (7)–(11)). Since the Hockett–Sherby law achieved the best fit overall until necking for Group I and II (cf. Table 3), it was used as a basis for derivation of a new mathematical expression. An extension of this law by power-law functions (cf. Equation (14)) showed a good fit to the experimental data of Group III.

(14) $$\sigma_{extended\;Hockett-Sherby}=\underset{Hockett–Sherby}{\underbrace{\sigma_{e}+\left(\sigma_{\infty }-\sigma_{e}\right)\left(1-e^{-m\cdot \varepsilon_{pl}^{n}}\right)}}+o\cdot \varepsilon_{pl}^{p}+q\cdot \varepsilon_{pl}^{r}$$

**Figure 5:**
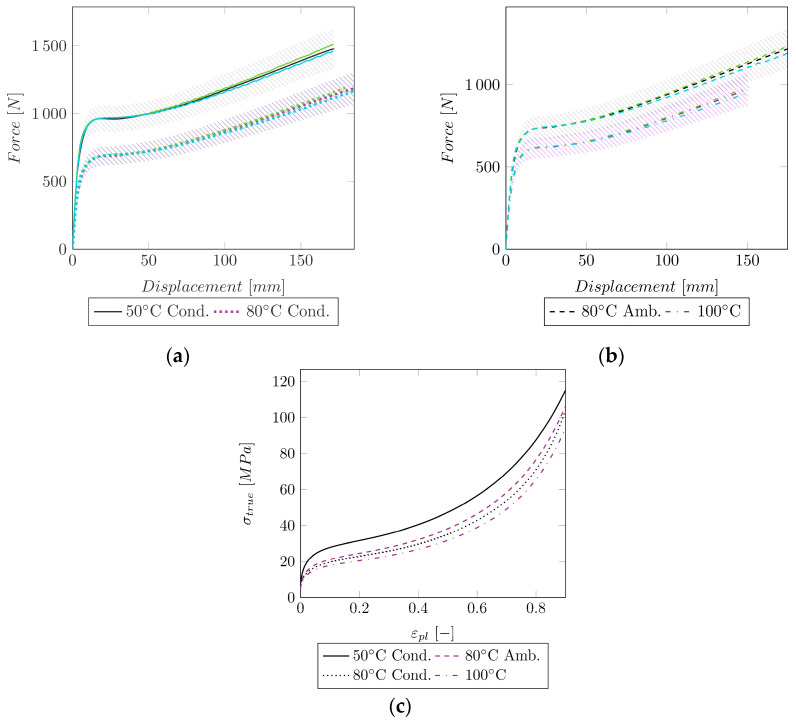
Comparison of the experimental data to the simulation of Group III for two mesh sizes. The shaded area indicates a ±10% force region around the experimental curve. Green: MS = 1 mm, Blue: MS = 2 mm. (**a**) Experimental and simulated force–displacement curves at 50 °C cond and 80°C cond; (**b**) experimental and simulated force–displacement curves at 80 °C ambient and 100 °C; (**c**) HCs for 50 °C (conditioned), 80 °C (ambient/conditioned), and 100 °C for an MS of 1 and 2 mm.

During the least squares fit, no negative values for the parameters ($\sigma_{\infty }$, m, n, o, p, q, r) were considered. The variables and the quality of the fit are shown in Table 4, and the resulting force–displacement curve is depicted in Figure 5. 

### 3.3. Curve Fitting Post-Necking

**Figure 6:**
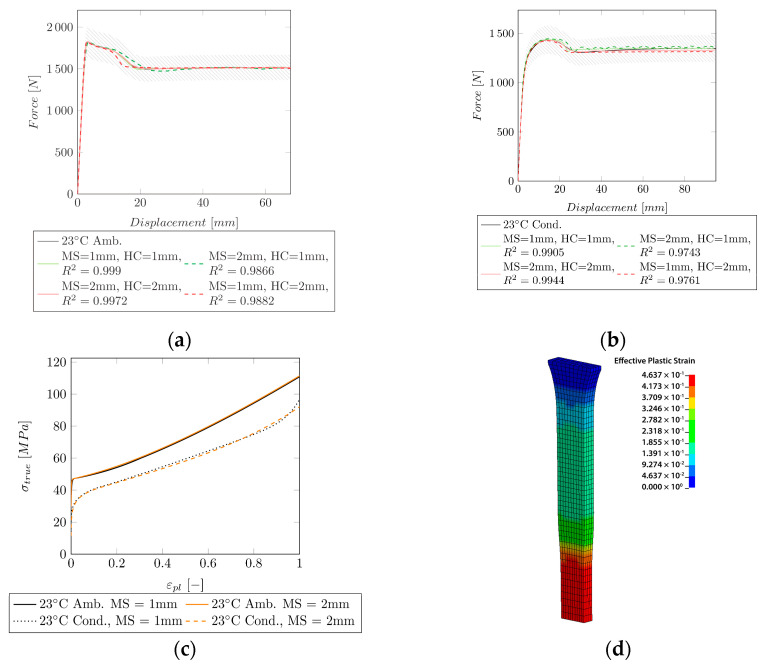
Comparison of the experimental data to the simulation of Group I for two mesh sizes (MS = 1 mm and MS = 2 mm). HC = 1 mm: HC identified with MS = 1 mm, HC = 2 mm: HC identified with MS = 2 mm. The shaded area indicates a ±10% force region around the experimental curve. (**a**) Experimental and simulated force–displacement curves at 23 °C (ambient) including the resulting $R^{2}$. (**b**) Experimental and simulated force–displacement curves at 23 °C (ambient) including the resulting $R^{2}$. (**c**) HCs for 23 °C (ambient/conditioned) for an MS of 1 and 2 mm. (**d**) Neck forming and propagation in the FE model.

For the fit of Group I (stable neck propagation) at 23 °C (ambient/conditioned), an inverse identification with a successive fit with Equation (12) or (13) was applied. The resulting force–displacement curves, the quality of the fit ($R^{2}$), and the experimental data are shown for the ambient and the conditioned samples in Figure 6a,b, respectively. For each mesh size (MS = 1 mm and MS = 2 mm) an HC (HC = 1 mm and HC = 2 mm) could be obtained (cf. Figure 6c) from the optimization routine. To show the impact on the result, the HC obtained with an MS of 1 mm was used for the simulation with an MS of 2 mm (MS = 1 mm, HC = 2 mm) and vice versa. Figure 6d shows the neck propagation, and the effective plastic strain in LS-DYNA.

**Figure 7:**
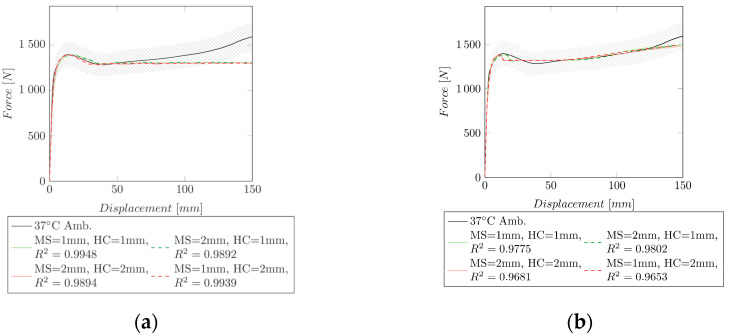

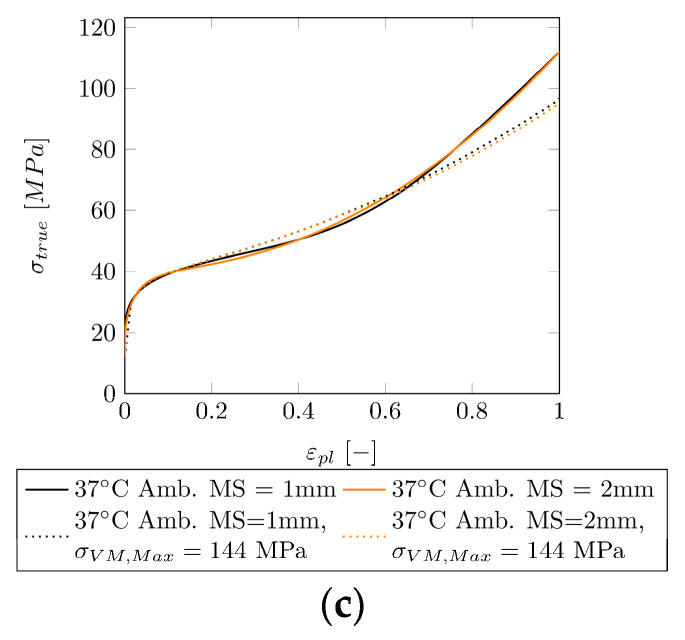
Comparison of the experimental data to the simulation of Group II for two mesh sizes (MS = 1 mm and MS = 2 mm). HC = 1 mm: HC identified with MS = 1 mm, HC = 2 mm: HC identified with MS = 2 mm. The shaded area indicates a ±10% force region around the experimental curve. (**a**) Experimental and simulated force–displacement curves at 37 °C (ambient) with the successive fitting of the HC including the resulting $R^{2}$. (**b**) Experimental and simulated force–displacement curves at 37 °C (ambient) with the extended Hockett–Sherby model including the resulting $R^{2}$. (**c**) HCs for the extended Hockett–Sherby model and the successive fit for an MS of 1 and 2 mm.

The same method (successive fit) was applied to Group II (necking followed by strain hardening) at 37 °C. However, no strain hardening could be achieved (cf. Figure 7a). Due to the missing strain hardening, the obtained HC was only valid until $\sigma_{VM}=144\;\mathrm{MPa}\;$. Furthermore, the extended Hockett–Sherby model, derived in Section 3.2, was used during the optimization to fit the entire force–displacement curve (cf. Figure 7b). The derived HCs can be seen in Figure 7c).

**Figure 8:**
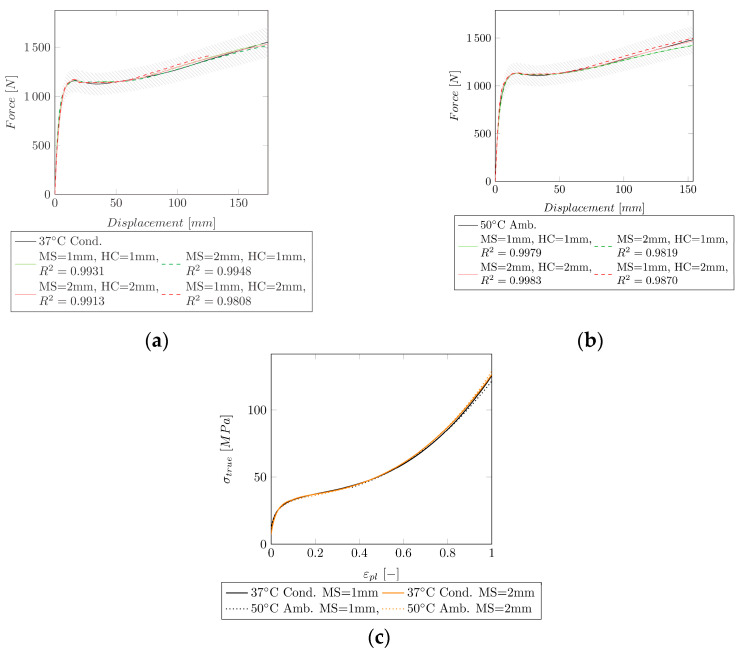
Comparison of the experimental data to the simulation of Group II for two mesh sizes (MS = 1 mm and MS = 2 mm). HC = 1 mm: HC identified with MS = 1 mm, HC = 2 mm: HC identified with MS = 2 mm. The shaded area indicates a ±10% force region around the experimental curve. (**a**) Experimental and simulated force–displacement curves at 37 °C (conditioned) with the extended Hockett–Sherby model including the resulting $R^{2}$. (**b**) Experimental and simulated force–displacement curves at 50 °C (ambient) with the extended Hockett–Sherby model including the resulting $R^{2}$. (**c**) HCs for the extended Hockett–Sherby model for an MS of 1 and 2 mm.

Due to the restrictions with the successive fit, only the extended Hockett–Sherby model was applied at 37 °C (conditioned) (cf. Figure 8a) and 50 °C (ambient) (cf. Figure 8b). The obtained HC can be seen in Figure 8c). 

The obtained HCs were expressed by using the extended Hockett–Sherby model. The variables are shown in Table 5. Due to the complex shape after the onset of necking till the start of neck propagation, the HC at 23 °C ambient (cf. Figure 6a) could not be fitted by the extended Hockett–Sherby model.

## 4. Discussion

The extraction of the HC until necking starts is state of the art, and many mathematical models are available to describe the curve. Five different models were compared against each other, and overall, the Hockett–Sherby model showed the best fit to the experimental data of the semi-crystalline polymer PA12. It must be noted that this model is only valid for a limited strain range. 

By extending the Hockett–Sherby model with two power-law functions, a perfect fit ($R^{2}=1$) to the experimental HC of Group III (no necking) could be achieved. Despite the perfect match to the experimental HC, a slight deviation to the experimental force–displacement curve could be observed. The simulation with a mesh size of 1 mm usually resulted in a slightly stiffer behavior, and a mesh size of 2 mm in a slightly less stiff behavior at fracture compared to the experimental data. However, this deviation appears mainly in the large strain region, where already a high mesh distortion is present. This in turn can lead to differences in the experimental data. Overall, a good fit for Group III could be achieved ($R^{2}>0.9927$).

The inverse identification could be applied to specimens in Group I (stable neck propagation) and Group II (necking followed by strain hardening). 

For Group I, a successive fit to the experimental data could be applied, and a good fit of $R^{2}=0.999\;$(MS = 1 mm, HC = 1 mm) and $R^{2}=0.9905\;$(MS = 1 mm, HC = 1 mm) could be obtained at 23 °C ambient and 23 °C conditioned, respectively. This method was not applicable for Group II, since no strain hardening could be obtained. At 37 °C (ambient) a nice fit of the necking could be obtained ($R^{2}=0.9948$, MS = 1 mm, HC = 1 mm). However, this model is only valid until $\sigma_{VM}$ = 144 MPa. A fit with the extended Hockett–Sherby model over the whole strain region was able to model the strain hardening. However, the accuracy in the strain region where necking takes place was reduced $(R^{2}=0.9775$). A reason, therefore, could be that the extended Hockett–Sherby model is not yet able to perfectly describe the complex behavior of the hardening curve, especially when strain hardening, and a large drop ratio are present. For the further fitting of Group II (37 °C conditioned ($R^{2}=0.9931$, MS = 1 mm, HC = 1 mm) and 50 °C ambient ($R^{2}=0.9979$, MS = 1 mm, HC = 1 mm)), only the extended Hockett–Sherby model was applied. Since both curves are similar, the obtained hardening curves are comparable. Comparing Group II to Group III specimens, it can be seen that the necking depends on the shape of the hardening curve. Although the same mathematical model was used for Group II and Group III, Group II exhibited necking, and Group III did not. 

By using two different mesh sizes, it could be shown that the mesh size influences the outcome of the hardening curve. When using the identified material model, the mesh size must be considered. By using a hardening curve that was identified with a different mesh size, $R^{2}$ was reduced by approximately 1%.

Since no measurement of the strain field on the specimen was available, the resulting strain field in LS-DYNA could not be compared to the experimental one. Therefore, the validation only relies on the force–displacement curve. DIC measurements could also aid in finding a mathematical model to fit hardening curves where necking is followed by strain hardening. During the experiment, the neck forms randomly along the parallel part of the dog bone. Even though the neck forms at different locations for each specimen the resulting force–displacement curves do not show increased scattering. Therefore, the location of the neck does not influence the result. During the FE simulation, necking always occurred in the middle part of the dog bone (cf. Figure 6d).

To obtain a temperature-dependent model for the linear–elastic material parameters, the model of Mahieux et al. [19] was applied. Even though a good fit for the Young’s modulus ($R^{2}>0.9994$) and the yield point ($R^{2}>0.99958$) could be achieved, and even though this model has been developed specifically for polymers, limited data were available for the fit. For a more reliable fit, more measurement points in the used temperature range (296–373 K) would be required. The currently identified parameters for this model might be correct for the measurement points at 23 °C, 37 °C, 50 °C, 80 °C, and 100 °C, but the behavior in-between these points still remains unknown. 

With the applied inverse identification approach, necking behavior can easily be fitted. If the necking is followed by a stable neck propagation, a successive fit to the experimental curve can be applied. Even though it is usually not recommended to fit too many parameters at once, an overall model is required if the curve is followed by strain hardening. Successive fits resulted in a stable neck propagation and not in the desired strain hardening. Furthermore, a successive fit requires a steady curve to avoid convergence issues. 

The shown inverse identification approach was applied to injection molded dog bone specimens since testing as well as simulation are already well established. In [18,30,31] it was shown that the mechanical properties vary between the specimen size, applied treatment, and the used manufacturing technology. This fact must be considered when using the proposed material properties. However, in the present work, the necessary methodology was developed and can be applied to different specimen types. Especially for use in PTCA balloon catheters that are manufactured from small, extruded tubing and that undergo various forming and heat treatment steps, this has to be considered. 

In the future, a mathematical model that also takes the temperature dependence of the plastic behavior into account would be favorable. 

Most available material models have been developed for metals. The yielding behavior of polymers may vary between tension and compression. Therefore, the Von Mises yield criterion is not fulfilled. Therefore, an inverse identification based on various tests and an appropriate yield criterion such as the Drucker–Prager criterion may be required. Furthermore, polymers often show a certain degree of anisotropy and no volume preservation in plasticity [13]. 

During this research, experimental data were available only for PA12. In the future, other polymers should be considered for the inverse identification approach. Furthermore, it would be of interest to see how well the extended Hockett–Sherby model represents the hardening of other polymers. 

## 5. Conclusions

Using a material model that goes beyond the linear elastic limit of materials can aid the development of high-performance polymeric products. The inverse identification approach shown in the present work can be helpful for the extraction of the appropriate material parameters, especially if only experimental force–displacement curves are available. Furthermore, the developed extended Hockett–Sherby model offers the possibility to use a single mathematical equation to define the hardening curve of semicrystalline polymers such as PA12. Finally, this works provides a set of material properties for the semicrystalline polymer PA12 under uniaxial tension in a temperature range of 23 °C–100 °C.

## Figures and Tables

**Table 1:** Linear elastic values. E and ν were taken from [18]. $\sigma_{e}$ represents the first value of the HC.

Temp (°C)	E (MPa)	$\sigma_{e}\;(\mathrm{MPa})$	ν (-)	E (MPa)	$\sigma_{e}\;(\mathrm{MPa})$	ν (-)
	Ambient			Conditioned		
23	1553.5	29.76	0.37	882.0	11.38	0.31
37	1036.3	11.86	0.44	641.5	7.67	0.46
50	606.2	7.26	0.46	446.4	5.32	0.46
80	315.2	4.69	0.47	297.6	4.42	0.47
100	272.1	4.04	0.47	-	-	-

**Table 2:** Variables of the mathematical model to fit the linear elastic values in a temperature range of 23–100 °C or 296–373 K.

Variable	Fit of E (MPa)		$\mathrm{Fit}\;\mathrm{of}\;\sigma_{e}\;(\mathrm{MPa})$	
	Ambient	Conditioned	Ambient	Conditioned
$P_{g}$	1952	1103	5857	18.57
$P_{r}$	395	310.0	4.722	4.575
$T_{d}$	401.3	411	408	408.8
$T_{g}$	312.8	312.7	200	302.1
m	21.17	19.13	4.328	15.96
n	12.8	18.16	21.2	22.84
$R^{2}$	0.9994	0.9995	0.99958	1

**Table 3:** Fitted variables of the Hockett–Sherby model until necking for 23 °C (Ambient and Conditioned), 37 °C (Ambient and Conditioned), and 50 °C (Ambient) $R^{2}$.

Temp. (°C)	Variable	$\sigma_{Hockett-Sherby}$	
		Amb	Cond
23 (valid up to $\sigma_{VM,amb}=47.5\;\mathrm{MPa}$, $\sigma_{VM,cond}=43\;\mathrm{MPa}$)	k	195.6	4.149
	n	0.8	0.4159
	$\sigma_{\infty }$	46.46	48.8
	$R^{2}$	0.996	0.9946
37 (valid up to $\sigma_{VM,amb}=41\;\mathrm{MPa}$, $\sigma_{VM,cond}=35\;\mathrm{MPa}$)	k	2.539	4.56
	n	0.3681	0.519
	$\sigma_{\infty }$	53.43	41.74
	$R^{2}$	0.9925	0.9997
50 (valid up to $\sigma_{VM,amb}=35\;\mathrm{MPa}$)	k	4.646	-
	n	0.5207	-
	$\sigma_{\infty }$	40.67	-
	$R^{2}$	0.9997	-

**Table 4:** Variables of the extended Hockett–Sherby model for Group III (50 °C Cond, 80 °C Amb/Cond, and 100 °C). $R^{2}$–HC is the goodness of the extended Hockett–Sherby model to the experimental HC. $R^{2}$–FD, MS = 1 mm is the goodness of the resulting force–displacement curve using a mesh size of 1 mm; and $R^{2}$–FD, MS = 2 mm is the quality of the resulting force–displacement curve using a mesh size of 2 mm.

Variable	50 °C Cond	80 °C Amb	80 °C Cond	100 °C
$\sigma_{\infty }$	30.98	24.52	25.08	20.66
m	7.389	6.309	4.429	6.088
n	0.5794	0.5784	0.5298	0.563
o	48.01	62.16	76.34	63.99
p	7.512	8.015	2.803	8.499
q	79.43	70.78	79.21	61.12
r	2.296	2.379	12.04	2.457
$R^{2}$–HC	1	1	1	1
$R^{2}$–FD, MS = 1 mm	0.994	0.9971	0.9961	0.9976
$R^{2}$–FD, MS = 2 mm	0.9958	0.9929	0.9937	0.9927

**Table 5:** Variables of the extend Hockett–Sherby model for 23 °C (conditioned), 37 °C (ambient/conditioned), and 50 °C (ambient).

Variable	23 °C Cond		37 °C Amb				**37 °C Cond**		**50 °C Amb**	
MS (mm)	1	2	$1\;(\sigma_{VM,}=144\;\mathrm{MPa})$	1	$2\;(\sigma_{VM}=144\;\mathrm{MPa})$	**2**	**1**	**2**	**1**	**2**
$\sigma_{\infty }$	34.96	39.9	43.42	37.88	41.72	32.2	39.40	36.26	36.06	37.57
m	23.36	6.967	4.961	3.395	5.908	14.5	5.94	14.45	8.047	6.316
n	3.494	0.4335	0.4241	4.579	0.4479	0.7373	0.57	0.7913	0.6062	0.5632
o	10.16	3.494	52.94	30.76	2.184	8.742	36.98	38.38	0.8628	90.57
p	4.373	0.7256	1.719	4.015	6.024	0.031366	2.79	2.385	4.171	2.661
q	41.96	88	0.4477	44.26	51.04	71.01	49.30	51.83	84.64	0.8859
r	1.232	2.596	8.613	0.2106	1.588	2.172	2.79	2.694	2.469	15.07

## Data Availability

The data presented in this study are available upon request from the corresponding author.

## Appendix A

To evaluate the convergence of the FE simulation, the results of three models with different mesh sizes (0.5 mm, 1 mm, and 2 mm) were compared using the material properties obtained from the experiment on dog bone specimens at 100 °C. In Figure A1, the result with a mesh size of 2 mm varies from the those with mesh sizes of 1 mm and 0.5 mm, respectively. However, simulations with mesh sizes of 1 mm and 0.5 mm showed the same results. From that it can be concluded that a mesh size of 1 mm is sufficient to obtain convergence.

## Appendix B

The results of the fit to Ludwik, Swift, Ghosh, and Voce (cf. Equations (7)–(10)) are shown in Table A1.

**Table A1:** Variables of fit to the Ludwik, Swift, and Ghosh models until necking for 23 °C (Ambient and Conditioned), 37 °C (Ambient and Conditioned), and 50 °C (Ambient) including the resulting $R^{2}$.

Temp. (°C)	Variable	$\sigma_{Ludwik}$		$\sigma_{Swift}$		$\sigma_{Ghosh}$		$\sigma_{Voce}$	
		Amb	Cond	Amb	Cond	Amb	Cond	Amb	Cond
$23\;(\mathrm{valid}\;\mathrm{up}\;\mathrm{to}\;\sigma_{VM,amb}=47.5\;\mathrm{MPa},\;$$\sigma_{VM,cond}=43\;\mathrm{MPa}$)	$\varepsilon_{0}$	−	−	1.155 × 10^−4^	2.92 × 10^−5^	2.34 × 10^−14^	2.34 × 10^−14^	−	−
	k	159.2	49.21	−	−	159.2	49.21	902.9	69.34
	n	0.426	0.2328	0.1149	0.1494	0.426	0.2328	−	−
	$\sigma_{\infty }$	−	−	−	−	−	−	15.46	29.3
	$R^{2}$	0.9629	0.9760	0.992	0.9923	0.9629	0.9760	0.9881	0.8632
$37\;(\mathrm{valid}\;\mathrm{up}\;\mathrm{to}\;\sigma_{VM,amb}=41\;\mathrm{MPa},\;$$\sigma_{VM,cond}=35\;\mathrm{MPa}$)	$\sigma_{e}$	−	−	4.23 × 10^−5^	1.193 × 10^−4^	2.34 × 10^−14^	2.33 × 10^−14^	-	−
	k	47.82	49.94	−	−	47.82	49.94	60.91	34.98
	n	0.2467	0.3017	0.1515	0.2156	0.2467	0.3017	-	-
	$\sigma_{\infty }$	−	−	−	−	−	−	27.36	27.31
	$R^{2}$	0.9852	0.9853	0.9959	0.9947	0.9852	0.9853	0.8319	0.9395
$50\;(\mathrm{valid}\;\mathrm{up}\;\mathrm{to}\;\sigma_{VM,amb}=35\;\mathrm{MPa}$)	$\sigma_{e}$	−	−	1.059 × 10^-4^	−	2.34 × 10^-14^	−	−	−
	k	48.86	−	−	−	48.85	−	34.9	−
	n	0.2987	−	0.2163	−	−	−	−	−
	$\sigma_{\infty }$	−	−	−	−	−	−	27.04	−
	$R^{2}$	0.9844	−	0.9941	−	0.9844	−	0.9396	−
